# Hippocampus and hypothermia: A missing link

**DOI:** 10.1111/dmcn.15427

**Published:** 2022-09-26

**Authors:** Paolo Montaldo, Sudhin Thayyil

**Affiliations:** ^1^ Centre for Perinatal Neuroscience Imperial College London London UK

While therapeutic hypothermia improves outcome after moderate and severe neonatal hypoxic‐ischaemic encephalopathy (HIE) in high‐income countries, many cooled infants without cerebral palsy (CP) have cognitive impairment and memory deficits during childhood.^1^ They are less school‐ready than typically developing peers and often have behavioural difficulties and special educational needs. These children may easily slip through the net if careful, long‐term follow‐up assessments are not performed. Despite the substantial health impact of these issues, significant knowledge gaps about the underlying mechanisms and therapeutic options remain.

Spencer et al.^2^ report additional childhood outcome and neuroimaging data from the Bristol cohort of 31 infants who had therapeutic hypothermia between 2007 and 2012, but did not have CP. On comparison with 32 typically developing age‐matched peers, the cooled infants had substantial cognitive and memory impairment at early school‐age. They also had lower whole‐brain grey and white matter volumes, hippocampal and thalamic volumes than typically developing peers. Cortical injury on neonatal magnetic resonance imaging (MRI) was associated with reduced volumes of hippocampi, thalami, grey matter, and white matter. While some of the analysis is exploratory, it is nevertheless important. These neuroimaging data add to the earlier reports of suboptimal cognition, working memory, and lower volume of mammillary bodies from this cohort while raising some important new questions.^1^, ^3^

Why does hippocampal injury persist in children without CP despite rescue hypothermic neuroprotection? Excluding children with CP suggests that this cohort of cooled infants were probably at the milder end of neonatal brain injury, where hypothermia is expected to be more neuroprotective. Hippocampus is one of the first regions to be injured in preclinical models of hypoxic–ischemic injury, well before deep brain nuclei injury.^4^ And yet no clear correlation of clinical condition at birth and injury to hippocampus or mammillary bodies is apparent. These issues are further complicated by the fact that hippocampus is not fully developed in humans before the age of 4 years; hence these injuries may be overlooked on neonatal MRI, especially when thicker slices are used.

Although preclinical models of single acute hypoxia‐ischemia have had a major role in the discovery of hypothermic neuroprotection, clinical scenario is far more heterogenous than current preclinical models. More complex preclinical models, representative of clinical scenario, are required to further advance the field of neuroprotection. Another major implication of these observations relates to the therapeutic drift of cooling in mild encephalopathy. Although CP is unlikely after mild encephalopathy, many of these infants have cognitive and memory deficits during childhood. Does it mean that hypothermia may not benefit these infants? Clearly, further research is required to explore these issues.

## Data Availability

Not required
